# A First Phenotypic and Functional Characterization of Placental Extracellular Vesicles from Women with Multiple Sclerosis

**DOI:** 10.3390/ijms22062875

**Published:** 2021-03-12

**Authors:** Serena Martire, Francesca Montarolo, Michela Spadaro, Simona Perga, Maria Ludovica Sforza, Luca Marozio, Federica Frezet, Stefania Bruno, Giulia Chiabotto, Maria Chiara Deregibus, Giovanni Camussi, Giovanni Botta, Chiara Benedetto, Antonio Bertolotto

**Affiliations:** 1 Neuroscience Institute Cavalieri Ottolenghi (NICO), Orbassano, 10043 Turin, Italy; spadaro_michela@yahoo.it (M.S.); simona.perga@unito.it (S.P.); ludovica.sforza96@gmail.com (M.L.S.); antonio.bertolotto@gmail.com (A.B.); 2 Neurology-CRESM (Regional Reference Center for Multiple Sclerosis), AOU San Luigi Gonzaga, Orbassano, 10043 Turin, Italy; 3 Department of Molecular Biotechnologies and Health Sciences, University of Turin, 10124 Turin, Italy; 4 Department of Neuroscience Rita Levi-Montalcini, University of Turin, 10124 Turin, Italy; 5 Department of Surgical Sciences, Obstetrics and Gynaecology, University of Turin, 10124 Turin, Italy; luca.marozio@unito.it (L.M.); federicafrezet@gmail.com (F.F.); chiara.benedetto@unito.it (C.B.); 6 Department of Medical Sciences and Molecular Biotechnology Center, University of Turin, 10124 Turin, Italy; stefania.bruno@unito.it (S.B.); giulia.chiabotto@unito.it (G.C.); giovanni.camussi@unito.it (G.C.); 7 2i3T Business Incubator and Technology Transfer, University of Turin, 10124 Turin, Italy; mariachiara.deregibus@unito.it; 8 Department of Pathology, Città della Salute e della Scienza di Torino, 10124 Turin, Italy; giovanni.botta@unito.it

**Keywords:** multiple sclerosis, extracellular vesicles, placenta, decidua, trophoblast, pregnancy

## Abstract

Pregnancy is a unique situation of physiological immunomodulation, as well as a strong Multiple Sclerosis (MS) disease modulator whose mechanisms are still unclear. Both maternal (decidua) and fetal (trophoblast) placental cells secrete extracellular vesicles (EVs), which are known to mediate cellular communication and modulate the maternal immune response. Their contribution to the MS disease course during pregnancy, however, is unexplored. Here, we provide a first phenotypic and functional characterization of EVs isolated from cultures of term placenta samples of women with MS, differentiating between decidua and trophoblast. In particular, we analyzed the expression profile of 37 surface proteins and tested the functional role of placental EVs on mono-cultures of CD14^+^ monocytes and co-cultures of CD4^+^ T and regulatory T (Treg) cells. Results indicated that placental EVs are enriched for surface markers typical of stem/progenitor cells, and that conditioning with EVs from samples of women with MS is associated to a moderate decrease in the expression of proinflammatory cytokines by activated monocytes and in the proliferation rate of activated T cells co-cultured with Tregs. Overall, our findings suggest an immunomodulatory potential of placental EVs from women with MS and set the stage for a promising research field aiming at elucidating their role in MS remission.

## 1. Introduction

Multiple sclerosis (MS) is a chronic inflammatory autoimmune disease of the central nervous system (CNS), leading to demyelination and axonal loss [1]. It is thought to arise from the interplay between genetic susceptibility and environmental exposure, which causes a breakdown of peripheral tolerance against CNS antigens [2]. MS is mainly driven by peripheral autoreactive CD4^+^ T lymphocytes, activated in the periphery and directed against CNS antigens. The reactivation of autoreactive T cells in the CNS triggers the recruitment and activation of additional immune cells to the areas of inflammation, the secretion of proinflammatory cytokines, chemokines and mediators, and the activation of resident microglia and astrocytes, which finally result in myelin damage [3].

Pregnancy is a naturally occurring disease modifier of MS, as pregnant patients experience a 70% reduction of the relapse rate in the third trimester and an increase in the post-partum [4,5]. This clinical improvement is ascribable to the establishment of a unique immune-tolerant condition induced by gestation, during which the maternal immune system adapt itself to tolerate the allogeneic fetus while maintaining the efficiency to fight against the external pathogens [6].

In physiological pregnancy, both local (at the maternal–fetal interface) and peripheral adaptations of the immune response have been described to occur, regarding both innate and adaptive immune cells [7,8,9]. Changes in the peripheral immune response mainly include activation of the innate immune system, a shift from proinflammatory T helper (Th) 1 and Th17 to anti- inflammatory Th2 immune response, and increased regulatory T (Treg) cells frequency [7]. The maternal–fetal interface is represented by the placenta, which comprises the maternally derived decidua and the fetally derived trophoblast [8]. Both sides of the placenta have been found to influence the function of the maternal immune cells by shedding extracellular vesicles (EVs) into the maternal circulation [10,11,12].

EVs are a heterogeneous population of cell-derived membrane vesicles which modulate the immune response the direct and indirect interaction with various immune cells. The placenta releases a wide variety of EVs, classified on the basis of their size and origin: exosomes (30–150 nm, from the endosomal pathway), microvesicles (150 nm–1 µm, from direct budding of the cell membrane), apoptotic bodies (1–5 µm, from cellular fragmentation of apoptotic cells), and syncytial nuclear aggregates (20–500 µm, clustering of nuclei shed from the syncytiotrophoblast plasma membrane) [10,11,12]. Approximately 20% of circulating exosomes in the gestating mother are from placental origin, and they progressively increase during gestation, achieving highest concentration in the third trimester [13,14]. EVs released from placenta also show distinct immunomodulatory properties based on gestational age [11]. During the first trimester, a major proinflammatory role of placental EVs have been described, to favor fetus implantation. In the second and third trimesters, EVs have been reported to inhibit peripheral blood mononuclear cells (PBMCs) proliferation, prevent lymphocytes activation and promote T cell apoptosis.

The immune-suppressive effects of pregnancy on experimental autoimmune encephalomyelitis (EAE), an MS murine model, have been associated to suppression of T cells mediated by serum exosomes [15,16]. Furthermore, the administration of human placental extract has been described to attenuate the neurological symptoms in the EAE model and enhance neuroprotection and myelin repair [17].

A better understanding of the mechanisms of fetal–maternal tolerance may help to clarify the biology of MS disease and provide inputs for the development of treatment strategies. In particular, the unique characteristics of EVs and their ability to deliver cargo to distant destinations in the body makes them ideal candidates as new therapeutic vectors. However, despite the interest aroused by this topic, the contribution of EVs in modulating the course of MS disease during pregnancy is almost unexplored.

This study provides a preliminary phenotypic and functional characterization of EVs secreted from placenta samples of women with MS and sets the stage for a research field aiming at elucidating their role in MS remission.

## 2. Results

### 2.1. Characteristics of MS and Healthy Control Groups

Women with MS and healthy control women (HC) were homogeneous regarding age at delivery, gestational age, birth weight of the child and type of delivery, as described in Table 1. Most of patients with MS have been receiving disease modifying treatment until the start of pregnancy. In particular, a total of six out of 15 women were under treatment with I line therapies, namely, interferon-β and glatiramer acetate, and one of them continued the therapy throughout the gestation. Six other women were under II line therapies, namely, natalizumab and fingolimog, and two of them continued the treatment throughout the first trimester. Finally, the three other women were untreated or at conception they had a wash out period longer than 6 months from previous therapies. A total of three out of 15 patients experienced a relapse in the second trimester of gestation.

### 2.2. Characterization of Placental EVs

**Figure 1:**
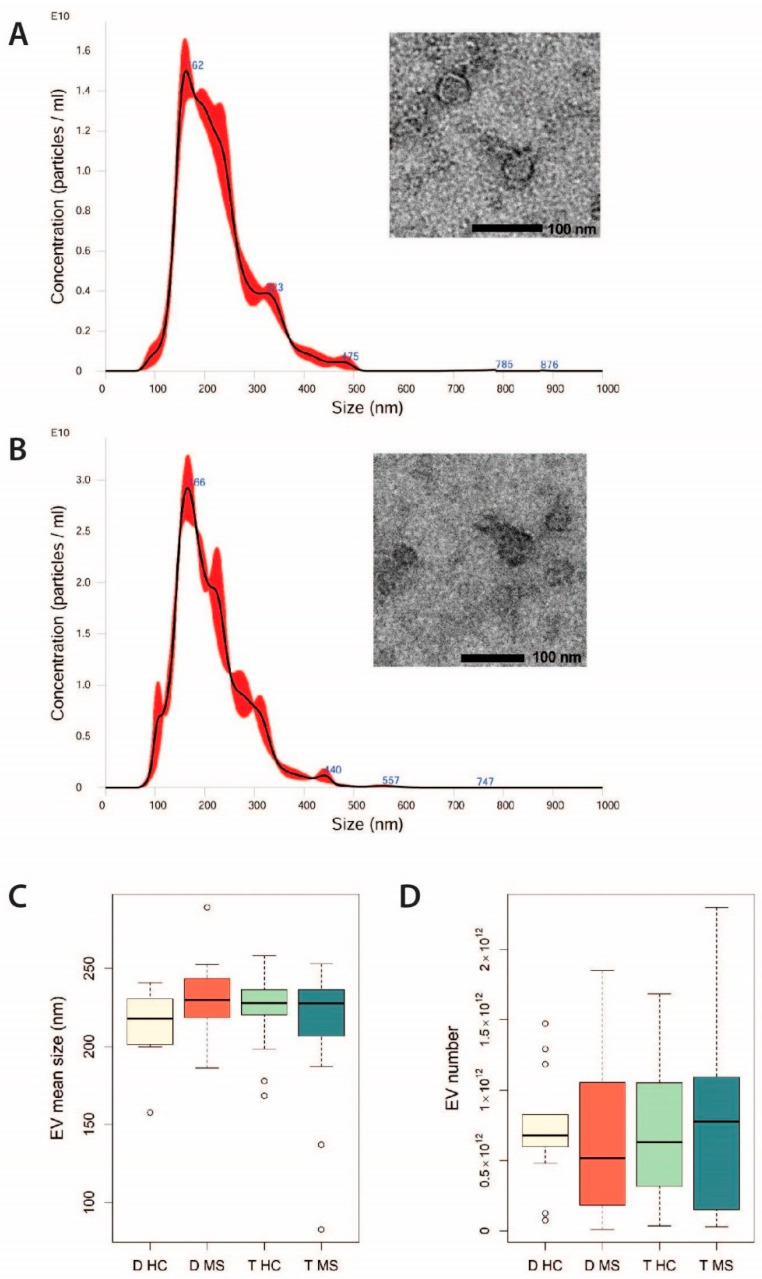
Characterization of placental extracellular vesicles (EVs). (**A**,**B**) NanoSight representative images of EVs obtained from the placenta of a HC (**A**) and of a patient with MS (**B**). A 405 nm laser light source was used to illuminate particles in the sample and the scattered light was captured and recorded by a camera. Three videos of 30 s were recorded to perform the analyses. The small inserts show representative micrographs of EVs analyzed by transmission electron microscopy and negatively stained with NanoVan (magnification, 100,000×). (**C**) Mean size and (**D**) quantity of EVs shed from decidua and trophoblast of women with MS and HC, obtained by NanoSight analysis. Quantity of EVs was normalized to the weight of the cultured tissue of origin.

EVs were isolated by ultracentrifugation from culture media of placental tissues. NanoSight analysis revealed a mean size of 214.8 ± 21.3 nm in samples from decidua of HC, 230.8 ± 24.8 nm in samples from decidua of patients with MS, 223.7 ± 24.8 nm in samples from trophoblast of HC, and 211.2 ± 45.5 nm in samples from trophoblast of patients with MS. The mean particle concentration per ml was 2.8 × 10^12^ ± 1.5 × 10^12^ in samples from decidua of HC, 1.4 × 10^12^ ± 1.5 × 10^12^ in samples from decidua of patients with MS, 3.9 × 10^12^ ± 2.4 × 10^12^ in samples from trophoblast of HC and 2.3 × 10^12^ ± 2.2 × 10^12^ in samples from trophoblast of patients with MS. Notably, the EV count was associated with the weight of the placenta sample (Pearson correlation coefficient r = 0.42, *p* = 0.0009). No significant differences were observed in the mean size and in the number of EVs secreted by decidua and trophoblast of women with MS and HC, normalized to the sample weight (Figure 1). Variability in the size of fixed Evs as seen by transmission electron microscopy was observed, so they were smaller than those detected by NanoSight analysis.

**Figure 2:**
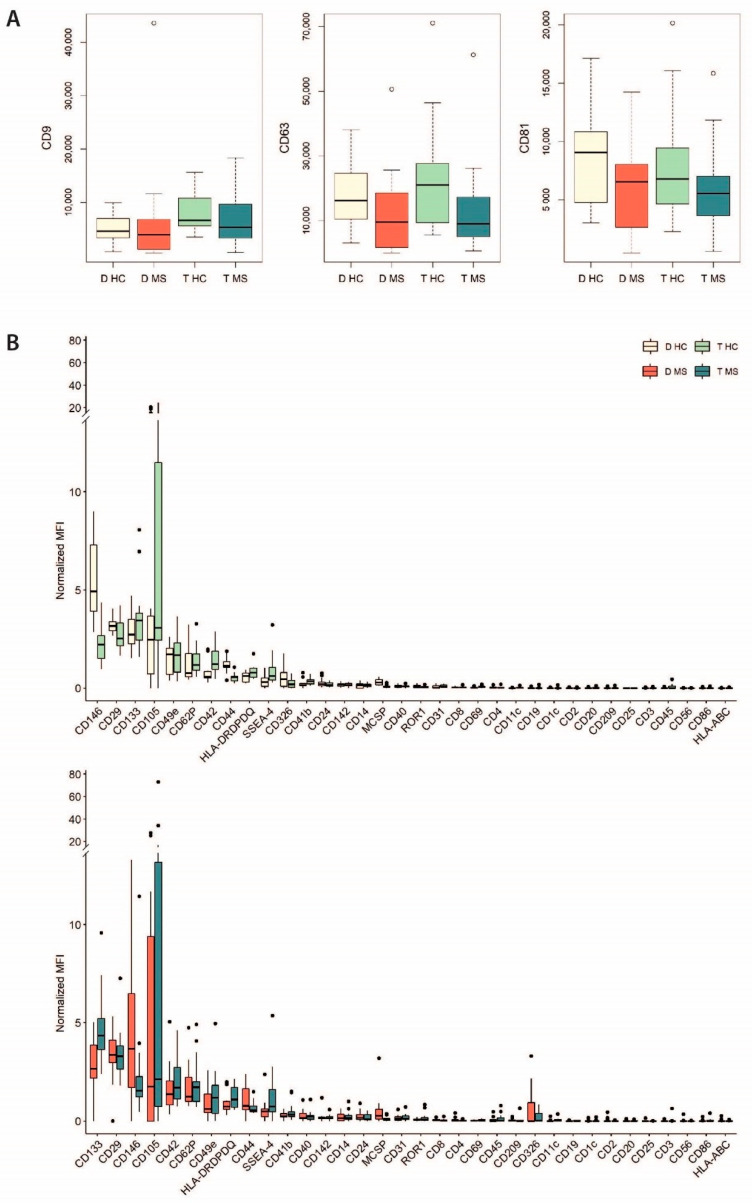
Characterization of surface markers of placental EVs by multiplex bead-based flow cytometry assay. (**A**) Median fluoresce intensity (MFI) of the exosomal tetraspanins CD9, CD63, and CD81. (**B**) MFI of the other markers relative to the mean value of tetraspanins’ MFI.

Evs were evaluated for the expression of 37 typical surface markers using the MACS multiplex bead-based flow cytometry assay [18,19,20]. Data analysis revealed that the Evs from all groups are positive for the exosomal tetraspanins CD9, CD63, and CD81 (Figure 2A). The expression level of the other markers was normalized to the mean expression levels of these tetraspanins, thus relative values are shown (Figure 2B). The most expressed markers, with a median relative expression greater than one in all the EV groups, were CD29, CD105, CD133, and CD146. On the other hand, the less expressed surface proteins, with a median relative expression equal to zero, were HLA-A/B/C, CD1c, CD2, CD3, CD19, CD20, CD25, CD56, and CD86.

**Figure 3:**
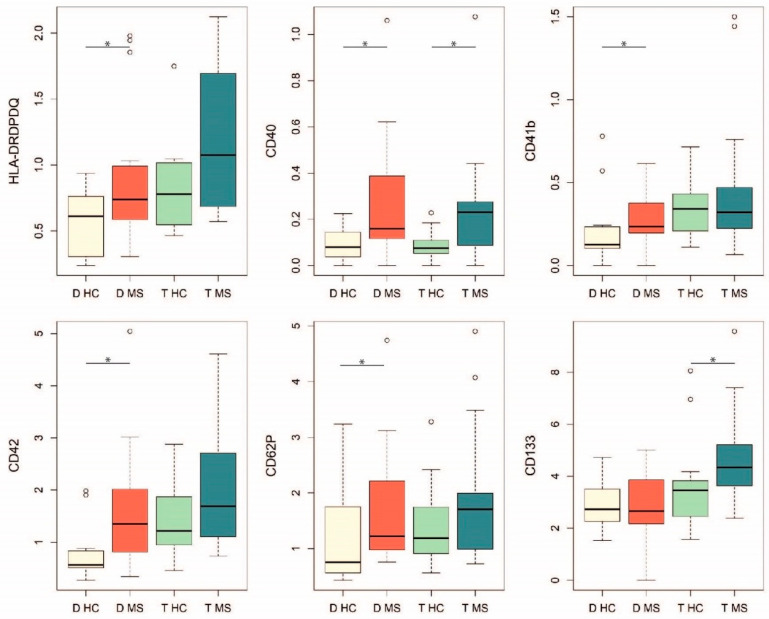
Surface markers differentially expressed between MS and HC groups. MFI of the marker relative to the mean value of tetraspanins’ MFI are represented. Mann–Whitney U test, * 0.01 ≤ *p* < 0.05.

The expression profile was compared between the MS and HC groups, and six differentially expressed markers were found (Figure 3). In particular, HLA-DR/DP/DQ, CD40, CD41b, CD42a, and CD62P expression was significantly higher in EVs from decidua samples of women with MS compared to HC (Mann–Whitney U test, *p* = 0.04, *p* = 0.03, *p* = 0.049, *p* = 0.01 and *p* = 0.04, respectively), while CD133 level was higher in EVs from trophoblast samples of women with MS compared to HC (Mann–Whitney U test, *p* = 0.04).

### 2.3. The Effect of EV Conditioning on LPS-Activated CD14^+^ Monocytes

**Figure 4:**
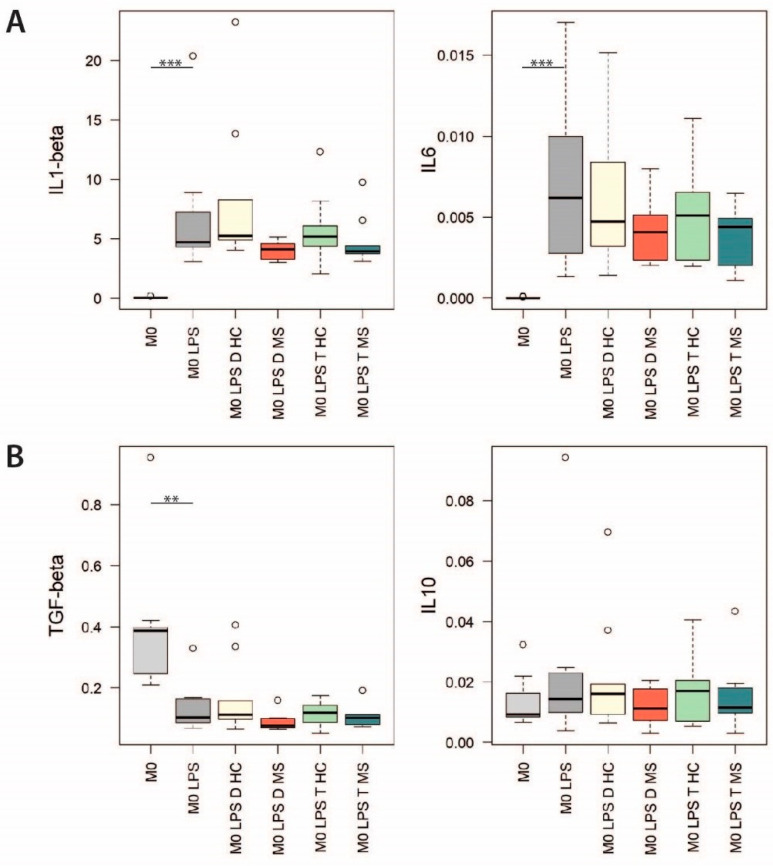
Gene expression analysis on activated CD14^+^ monocytes conditioned with EVs. Relative expression (2^−∆Ct^) of (**A**) the proinflammatory cytokines IL1-β and IL-6 and (**B**) the anti-inflammatory cytokines TGF-β and IL-10. Kruskal–Wallis with Dunn’s post hoc test, ** 0.001 ≤ *p* < 0.01; *** *p* < 0.001.

The transcript level of a panel of proinflammatory (IL1-β and IL-6) and anti-inflammatory (TGF-β and IL-10) cytokines was compared in LPS-activated CD14^+^ monocytes monocultures conditioned or not with EVs (Figure 4). The expression level of cytokines did not change significantly following conditioning with any type of EVs (Kruskal–Wallis with Dunn’s post hoc test, *p* > 0.05). However, the conditioning with EVs from both decidua and trophoblast samples of women with MS was associated with a greater decrease in the median expression level of proinflammatory cytokines.

### 2.4. The Effect of EV Conditioning on Tregs Activity

**Figure 5:**
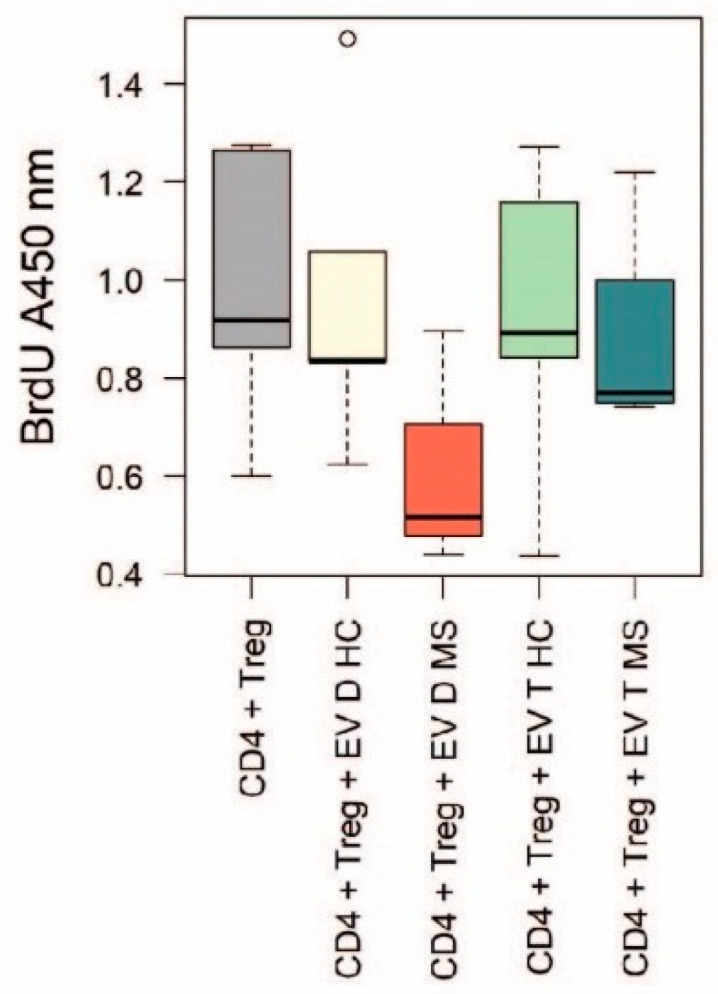
BrdU cell proliferation assay on co-cultures of CD4^+^ T cells activated via CD3/CD28 and CD4^+^CD25^+^CD127^dim/−^ Tregs conditioned or not with EVs. Kruskal–Wallis test, *p* = 0.44.

The modulatory potential of EVs on the inhibitory activity of CD4^+^CD25^+^CD127 ^dim/−^ Tregs was tested by comparing the proliferation rate of CD4^+^ T cells activated via CD3 and CD28 co-cultured with conditioned or unconditioned Tregs. No significant differences between groups were found (Kruskal–Wallis test, *p* = 0.44). However, the conditioning with EVs from decidua samples of women with MS was associated with a greater decrease in the median proliferation rate of T cells (Figure 5).

## 3. Discussion

Pregnancy is a unique situation of physiological immunomodulation, as well as a strong MS disease modulator, whose mechanisms are still poorly understood [4,5,6]. Information exchange between the mother and the fetus is crucial to establish and maintain a healthy pregnancy. Particles released from placenta, in particular EVs, are thought to have a key role in this communication and in the pregnancy-induced immunomodulation [10,11]. Studies of the potential function of EVs of placental origin during MS pregnancy are lacking. However, the administration of human placental extract and of serum exosomes from pregnant mice have been shown to reduce disease severity in the EAE model [16,17].

Here, we provide, for the first time to our knowledge, a preliminary phenotypic and functional characterization of EVs secreted from term placenta samples of women with MS, differentiating between EVs of decidua and trophoblast origin. The sample size is limited, due to the logistical difficulties related to the collection of this type of sample and given the exploratory nature of the study. Our analysis does not take into account the dynamic changes in the immunomodulatory potential of placental EVs that may occur during the entire course of pregnancy. However, it describes the scenario at the end of the third trimester, during which patients usually experience the greatest reduction in disease activity.

We isolated EVs from culture media of placental tissues by ultracentrifugation, which still represent the golden standard technique [21]. As expected, EV count correlated with weight of the cultured tissue. After adjusting for this factor, we observed similar count and size distribution of EVs from decidua and trophoblast samples of HC and women with MS. Fixed EVs, as seen by transmission electron microscopy, were smaller than those detected by NanoSight analysis, may be due to the influence of temperature and Brownian motion incorporated into the NanoSight method of EV characterization.

We also characterized the EV phenotype by the analysis of a panel of 37 surface proteins. All the four groups of EVs were positive for the typical markers of embryonic, mesenchymal and hematopoietic stem cells, such as CD133, CD29, CD146, and CD105, which showed the highest expression level, and also CD44, SSEA-4, CD326, and CD24 [22,23,24]. Notably, a growing body of evidence describes EVs as the primary mechanism of intercellular communication between stem cells and immune cells [25,26]. Particularly, EVs derived from stem/progenitor cells have been reported to inhibit proliferation and activation of immune cell and promote immune tolerance [25,26]. In this context, a role of placental EVs in the stem cells-microbiome connection could also be hypothesized [27,28]. On the other hand, almost all EV samples were negative for several surface proteins expressed by antigen-presenting cells (HLA-A/B/C molecules, CD86, and CD1c), mature or activated T cells (CD2, CD3, CD25), activated immune cells with cytotoxic properties (CD56), and B cells (CD19, CD20). Dendritic cells (DCs), a class of antigen-presenting cells, constitute about 1% of the total decidual immune cells throughout gestation and mainly display an immature phenotype, characterized by low expression of CD40, CD80, CD86, and CD205 [29,30]. Although the contribution of DC-derived EVs to maternal tolerance is still not completely elucidated, it is known that EVs derived from immature DCs, unlikely those from mature DCs bearing CD80 and CD86, do not induce proliferation of naïve CD4^+^ T cells [31]. Few studies have investigated changes in the frequency of T and B cells during pregnancy, particularly in women with MS, and with contradictory results. However, our finding could reflect a decrease in these cell subsets [32].

Some differences emerged in the expression profile of surface markers between the MS and HC groups. In EVs from samples of women with MS, particularly of decidua origin, we observed a higher expression of specific molecules engaged in antigen presentation and T cell stimulation, i.e., HLA Class II molecules, and CD40, and in cell adhesion and platelets aggregation, i.e., CD41b, CD42a, and CD62P. An increased level of platelet-derived EVs has been reported in inflammatory associated disorders, potentially reflecting the persistence of a pathological condition in pregnant women with MS, although in a remission phase [33]. However, besides being considered strong proinflammatory mediators, platelet-derived EVs have also been recently described to have an anti-inflammatory role [33]. CD133 was further expressed at higher levels in EVs from trophoblast samples of women with MS compared to HC. This finding is of particular interest, considering that stem/progenitor cells derived EVs, and particularly CD133^+^ cell-derived EVs, have been shown to repair kidney, nervous system, and heart tissues after injury in various disease models [34,35,36,37]. However, implications in immunomodulatory mechanisms of MS need to be investigated.

Along with the phenotypic characterization, we performed a preliminary functional characterization of placental EVs, aimed at evaluating their modulatory role on mono-cultures of monocytes and co-cultures of CD4^+^ T and Treg cells. Considering the exploratory nature of the study and the limited sample size of placenta donors, we carried out functional experiments only on two PBMCs donors. This enabled to prioritize the immunomodulatory potential of EVs over the interindividual variability in the immune cells response, but it also represents a major limitation of the study. We observed that the conditioning of activated monocytes with EVs from samples of women with MS, both decidua and trophoblast, is associated with a decrease in the expression level of proinflammatory cytokines, although not statistically significant. In addition, we observed a decrease in the proliferation rate of activated CD4^+^ T cells co-cultured with Tregs conditioned with EVs from decidua samples of women with MS, but still not significant. This could be due to an inadequate statistical power; however, it could also be interpreted in light of recent evidence from the literature which have reported that CD14^+^ cells are the major recipient cell subset of EVs and have a crucial role in mediating their immunomodulatory functions [38,39]. In fact, it has been shown that EVs from regenerative cardiac cells fail to modulate proliferation of activated CD3^+^ T cell monocultures. On the other hand, the EV-conditioning of PBMCs cultures, or the co-culture of EV-conditioned CD14^+^ cells with activated CD3^+^ T cells, led to a significant reduction of T cell proliferation as well as to an increased proportion of Tregs [38]. Conditioning of specific immune cell types, such as CD14^+^ cells, could therefore be an essential step in the EV-induced inhibition of T cell proliferation and could explain why we observed a moderate effect in our functional assays.

Overall, our findings suggest a potential immunomodulatory role of placental EVs from women with MS and unveil some differences in their phenotype and functions compared to healthy women. Further studies on cell cultures and animal models, as well as the investigation of the EV molecular cargo, are required to unravel the mechanisms whereby placental EVs exert their beneficial effects on dysfunctional immune systems, and to direct future therapeutic interventions for patients with MS and other autoimmune diseases.

## 4. Materials and Methods

### 4.1. Sample Collection

Placental samples from 15 women with relapsing-remitting (RR) MS and 15 HC were collected immediately after the delivery at the Department of Surgical Sciences, Obstetrics and Gynaecology, University of Turin, between November 2018 and October 2019. Decidua and trophoblast have been macroscopically separated and subsequently transported at 4 °C to the Neurobiology laboratory of the Neuroscience Institute Cavalieri Ottolenghi (NICO) within 12 h for further processing. The study was approved by the ethical committee of Città della Salute e della Scienza di Torino (0067257/2018). All the women enrolled gave their written informed consent.

### 4.2. EVs Isolation

Placental samples were weighted, manually shattered with a scalpel, and cultured in vitro for 24 h in RPMI 1640 medium (Invitrogen Life Technologies, Carlsbad, CA, USA) 10% heat-inactivated ultracentrifuged FBS (Invitrogen Life Technologies, Carlsbad, CA, USA) at 37 °C and 5% CO_2_. The culture medium was collected and subjected to subsequent centrifugation (1000× *g* for 10 min 4 °C, 2000× *g* for 20 min 4 °C, and 10,000× *g* for 30 min 4 °C) to remove cellular debris. Supernatant was ultracentrifuged in a type 70 TI fixed-angle titanium rotor (Beckman Coulter, Brea, CA, USA) at 100,000× *g* at 4 °C for 2 h. The pellet was suspended in PBS 1% DMSO (Sigma-Aldrich, St Louis, MO, USA) and stored at −80 °C for later use.

### 4.3. Characterization of EVs by Nanoparticle Tracking Analysis (NTA)

Size distribution and concentration of EVs were measured using NanoSight LM10 instrument (Malvern Instruments, Malvern, UK). A 405 nm laser light source was used to illuminate particles in the sample and the scattered light was captured and recorded by a camera. Three videos of 30 s were recorded to perform the analyses. Data were analyzed with the Nanosight NTA Software version 3.1 (Malvern Instruments, Malvern, UK): this software automatically tracked and sized the particles based on the Brownian motion and the diffusion coefficient and converted this information into size and concentration parameters, using the Stokes–Einstein equation.

### 4.4. Characterization of EVs by Electronic Microscopy

Transmission electron microscopy was performed on Evs isolated by ultracentrifugation on 200 mesh nickel formvar carbon-coated grids (Electron Microscopy Science, Hatfield, PA, USA) and left to adhere for 20 min, as previously described [40]. The grids were then incubated with 2.5% glutaraldehyde containing 2% sucrose and after washings in distilled water the Evs were negatively stained with NanoVan (Nanoprobes, Yaphank, NK, USA) and observed using a Jeol JEM 1010 electron microscope (Jeol, Tokyo, Japan).

### 4.5. Characterization of EV Surface Markers by Flow Cytometry

The expression of 37 surface markers (CD1c, CD2, CD3, CD4, CD8, CD9, CD11c, CD14, CD19, CD20, CD24, CD25, CD29, CD31, CD40, CD41b, CD42a, CD44, CD45, CD49e, CD56, CD62P, CD63, CD69, CD81, CD86, CD105, CD133, CD142, CD146, CD209, CD326, HLA-ABC, HLA-DRDPDQ, MCSP, ROR1, and SSEA-4) was evaluated using the human cytofluorimetric bead-based MACSPlex exosome kit (Miltenyi Biotec, Bergish Gladbach, Germany) according to manufacturer’s protocol as previously described [18,19,20]. Flow cytometric analysis was performed using the Cytoflex flow cytometer (Beckman Coulter, Brea, CA, United States). The median fluorescence intensity (MFI) values of each marker were corrected for background signal by subtracting the respective MFI values from the non-EV containing buffer samples included in every session of analysis. Finally, the MFI values were normalized to the mean value of the CD9/CD63/CD81 tetraspanins’ MFI, in order to determine the relative levels of each marker.

### 4.6. Isolation of CD14^+^ Monocytes, CD4^+^ T Cells and CD4^+^CD25^+^CD127^dim/−^ Tregs

PBMCs were isolated from buffy coats of healthy donors by density gradient centrifugation using Lymphoprep™ solution (07801, STEMCELL Technologies Inc.,Vancouver, BC, Canada). CD14^+^ monocytes, CD4^+^ T cells, and CD4^+^CD25^+^CD127^dim/−^ Tregs were separated with magnetic-based isolation kits using the autoMACS Pro Separator station (Miltenyi Biotec, Bergish Gladbach, Germany) according to manufacturer’s protocol.

CD14^+^ MicroBeads (130-096-533, Miltenyi Biotec, Bergish Gladbach, Germany) were used to separate CD14^+^ monocytes via positive selection, CD4^+^ T cell Isolation Kit (CD4^+^ T cell Microbead Cocktail, CD4^+^ T cell Biotin-Antibody Cocktail, human, Miltenyi Biotec, Bergish Gladbach, Germany) to separate CD4^+^ T cells via negative selection and CD4^+^CD25^+^CD127^dim/−^ regulatory T cells isolation kit II (human, 130-094-775, Miltenyi Biotec, Bergish Gladbach, Germany) to separate Tregs via pre-enrichment of CD4^+^CD127^dim/−^ and subsequent positive selection of CD4^+^CD25^+^CD127^dim/−^.

### 4.7. Monoculture of CD14^+^ Monocytes Conditioned with EVs

For this experiment, CD14^+^ cells from 2 healthy donors and Evs from 10 HC and 10 women with MS were used. CD14^+^ cells were cultured at a concentration of 1 × 10^6^/mL in RPMI 1640 medium containing 10% heat-inactivated ultracentrifuged FBS, 1% penicillin/streptomycin (Invitrogen Life Technologies), and 1% glutamine (Invitrogen Life Technologies, Carlsbad, CA, USA) (complete medium) for 24 h at 37 °C and 5% CO_2_ in two separate flasks, in presence or not of LPS (10 ng/mL). Subsequently, CD14^+^ monocytes (M0) were transferred in 6-well plates, at a concentration of 2 × 10^6^ cells/well in 2 ml of complete medium. M0 stimulated with LPS were conditioned with EVs shed by decidua and trophoblast from women with MS and HC, at a concentration of 5000 EV/cell. Finally, six experimental conditions were obtained: (1) M0; (2) M0 + LPS; (3) M0 + LPS + pEV D HC; (4) M0 + LPS + pEV D MS; (5) M0 + LPS + pEV T HC; (6) M0 + LPS + pEV T MS. Each condition was plated in duplicate. After 24 h of incubation, duplicate wells were merged and 4 × 10^6^ CD14^+^ cells for each condition were collected, centrifuged 300× *g* for 15 min at 4 °C and stored at −80 °C.

### 4.8. RNA Extraction from CD14^+^ Monocytes and Gene Expression Analysis

Total RNA was extracted from the cell pellet using the Maxwell^®^ RSC Instrument (Promega, Madison, Wisconsin, USA) with Maxwell^®^ RSC miRNA Tissue Kit (AS1460, Promega, Madison, Wisconsin, USA) according to manufacturer’s protocol, and stored at −80 °C. RNA was quantified using the Tecan INFINITE^®^ 200 Pro multiplate reader (Tecan Life Sciences, Männedorf, Switzerland) and reverse transcribed at a final concentration of 20 ng/μL using the RT High Capacity kit (Thermo Fisher Scientific Inc., Waltham, MA, USA) following manufacturer’s protocol.

The expression level of IL1-β, IL-6, TGF-β, and IL-10 was analyzed by real-time PCR using TaqMan reagents (Thermo Fisher Scientific Inc., Waltham, MA, USA) and the StepOne Plus instrument (Thermo Fisher Scientific Inc., Waltham, MA, USA). The expression levels of target genes were calculated by the normalized comparative cycle threshold (Ct) method (2^−ΔCt^) using glyceraldehyde 3-phosphate dehydrogenase (GAPDH) as the reference gene.

### 4.9. Co-Culture of CD4^+^ T Cells and CD4^+^CD25^+^CD127^dim/−^ Tregs Conditioned with EVs

For this experiment, cells from 2 healthy donors and EVs from 5 HC and 4 women with MS were used. After isolation, CD4^+^ T cells were activated via CD3 and CD28 using 1 μL/1 × 10^5^ cells of T Cell TransAct™ (TA, human, 130-111-160, Miltenyi Biotec., Bergish Gladbach, Germany) and cultured in a 96-well plate at a concentration of 1 × 10^6^/mL in complete medium for 24 h at 37 °C and 5% CO_2_. In parallel, 5 fractions of Tregs were cultured for 24 h in 4-well plates at a concentration of 5 × 10^5^/mL in complete medium; four of them were conditioned with EVs shed by decidua and trophoblast from women with MS and HC, at a concentration of 10,000 EV/cell. After, Tregs were co-cultured with CD4^+^ T cells at a 1:2 ratio in the 96-well plate for 4 days at a final volume of 300 μL/well. Finally, five experimental conditions were obtained: (1) CD4^+^+ TA + Tregs; (2) CD4^+^+ TA + Tregs + EV D HC; (3) CD4^+^+ TA + Tregs + EV D MS; (4) CD4^+^+ TA + Tregs + EV T HC; (5) CD4^+^+ TA + Tregs + EV T MS. Each condition was prepared in triplicate.

### 4.10. Proliferation Assay of CD4^+^ T Cells-Tregs Co-Cultures

Proliferation rates of the five experimental conditions were assessed by the Bromodeoxyuridine (BrdU) cell proliferation ELISA colorimetric kit (Abcam, ab126556, Cambridge, UK), following manufacturer’s protocol and using Tecan INFINITE^®^ 200 Pro multiplate reader, set at a dual wavelength of 450/550 nm (Tecan Life Sciences, Männedorf, Zürich, Switzerland).

### 4.11. Statistical Analysis

Statistical analysis was performed using the R Software version 4.0.2 (R Core Team (2020), Vienna, Austria, www.r-project.org, accessed on 23 February 2021). Normality of distributions was verified with Shapiro–Wilk test. Pearson correlation was used to evaluate the association between EV count and weight of the placenta samples. Differences among groups were analysed with Mann–Whitney or Wilcoxon signed ranks test or Kruskal–Wallis followed by Dunn’s post hoc test, as appropriate. *p* values were adjusted for multiple comparisons using the Benjamini–Hochberg method. *p* values < 0.05 were considered statistically significant.

## Figures and Tables

**Table 1:** Characteristics of the women cohorts.

Characteristics	MS	HC	*p* Values
Maternal age, years	33.3 (5.5)	32.9 (6.7)	0.84 ^a^
Gestational age, weeks	37.0 (2.7)	38.2 (1.7)	0.18 ^a^
Birth weight, grams	2791.4 (661.5)	3147.9 (561.7)	0.14 ^a^
Cesarean section	8 (53)	12 (80)	0.24 ^b^

Data are presented as mean (SD) or number (%). ^a^ *t*-test; ^b^ Fisher’s exact test.

## Abbreviations

EAE	Experimental autoimmune encephalomyelitis
EV	Extracellular vesicles
HC	Healthy control
M0	CD14^+^ monocytes
MFI	Median fluorescence intensity
MS	Multiple sclerosis
PBMC	Peripheral blood mononuclear cell
Th	T helper
Treg	Regulatory T cell
